# Functional Impairment in Behavioral Variant Frontotemporal Dementia: Cognitive, Behavioral, Personality, and Brain Perfusion Contributions

**DOI:** 10.3390/jpm15100466

**Published:** 2025-10-01

**Authors:** Electra Chatzidimitriou, Georgios Ntritsos, Roza Lagoudaki, Eleni Poptsi, Emmanouil Tsardoulias, Andreas L. Symeonidis, Magda Tsolaki, Eleni Konstantinopoulou, Kyriaki Papadopoulou, Panos Charalambous, Katherine P. Rankin, Eleni Aretouli, Chrissa Sioka, Ioannis Iakovou, Theodora Afrantou, Panagiotis Ioannidis, Despina Moraitou

**Affiliations:** 1 Department of Cognition, Brain and Behavior, School of Psychology, Faculty of Philosophy, Aristotle University of Thessaloniki (AUTh), 54124 Thessaloniki, Greece; electra_hatzidimitriou@hotmail.com (E.C.); eledimkon@psy.auth.gr (E.K.); demorait@psy.auth.gr (D.M.); 2 2nd Department of Neurology, AHEPA University Hospital, Aristotle University of Thessaloniki (AUTh), 54636 Thessaloniki, Greece; afrantou@gmail.com (T.A.); ispanagi@auth.gr (P.I.); 3 Laboratory of Neurodegenerative Diseases, Center for Interdisciplinary Research and Innovation, Aristotle University of Thessaloniki (CIRI-AUTh), 57001 Thessaloniki, Greece; poptsi.e@alzheimer-hellas.gr (E.P.); tsolakim1@gmail.com (M.T.); 4 Department of Economics, School of Economics and Management Sciences, University of Ioannina, 45110 Ioannina, Greece; gntritsos@uoi.gr; 5 Laboratory of Physiology, School of Medicine, Aristotle University of Thessaloniki (AUTh), 54124 Thessaloniki, Greece; 6 Greek Association of Alzheimer’s Disease and Related Disorders (GAADRD), Petrou Sindika 13 Str., 54643 Thessaloniki, Greece; 7 School of Electrical and Computer Engineering, Faculty of Engineering, Aristotle University of Thessaloniki (AUTh), 54124 Thessaloniki, Greece; etsardou@ece.auth.gr (E.T.); asymeon@eng.auth.gr (A.L.S.); 8 2nd Academic Department of Nuclear Medicine, AHEPA University Hospital, Aristotle University of Thessaloniki (AUTh), 54636 Thessaloniki, Greece; kikipap97@gmail.com (K.P.); charalambous.pa@hotmail.com (P.C.); iiakovou@auth.gr (I.I.); 9 Memory and Aging Center, Department of Neurology, University of California, San Francisco, CA 94158, USA; kate.rankin@ucsf.edu; 10 Department of Psychology, School of Social Sciences, University of Ioannina, 45110 Ioannina, Greece; earetouli@uoi.gr; 11 Department of Nuclear Medicine, University Hospital of Ioannina, 45500 Ioannina, Greece; csioka@uoi.gr

**Keywords:** bvFTD, functional impairment, correlates, cognitive decline, behavioral disturbances, personality changes, brain perfusion, SPECT imaging

## Abstract

**Background/Objectives:** Behavioral variant frontotemporal dementia (bvFTD), the most prevalent clinical subtype within the frontotemporal lobar degeneration spectrum disorders, is characterized by early and prominent changes that significantly disrupt everyday functioning. This study aims to identify the key correlates of functional status in bvFTD by investigating the relative contributions of cognitive deficits, behavioral disturbances, personality changes, and brain perfusion abnormalities. Additionally, it seeks to develop a theoretical framework to elucidate how these factors may interconnect and shape unique functional profiles. **Methods:** A total of 26 individuals diagnosed with bvFTD were recruited from the 2nd Neurology Clinic of “AHEPA” University Hospital in Thessaloniki, Greece, and underwent a comprehensive neuropsychological assessment to evaluate their cognitive functions. Behavioral disturbances, personality traits, and functional status were rated using informant-based measures. Regional cerebral blood flow was assessed using Single Photon Emission Computed Tomography (SPECT) imaging to evaluate brain perfusion patterns. Penalized Least Absolute Shrinkage and Selection Operator (LASSO) regression analysis was performed to identify the most robust correlates of functional impairment, followed by path analyses using structural equation modeling to explore how these factors may interrelate and contribute to functional disability. **Results:** The severity of negative behavioral symptoms (e.g., apathy), conscientiousness levels, and performance on neuropsychological measures of semantic verbal fluency, visual attention, visuomotor speed, and global cognition were identified as the strongest correlates of performance in activities of daily living. Neuroimaging analysis revealed hypoperfusion in the right prefrontal (Brodmann area 8) and inferior parietal (Brodmann area 40) cortices as statistically significant neural correlates of functional impairment in bvFTD. Path analyses indicated that reduced brain perfusion was associated with attentional and processing speed deficits, which were further linked to more severe negative behavioral symptoms. These behavioral disturbances were subsequently correlated with declines in global cognition and conscientiousness, which were ultimately associated with poorer daily functioning. **Conclusions:** Hypoperfusion in key prefrontal and parietal regions, along with the subsequent cognitive and neuropsychiatric manifestations, appears to be associated with the pronounced functional limitations observed in individuals with bvFTD, even in early stages. Understanding the key determinants of the disease can inform the development of more targeted, personalized treatment strategies aimed at mitigating functional deterioration and enhancing the quality of life for affected individuals.

## 1. Introduction

Behavioral variant frontotemporal dementia (bvFTD) is the most prevalent clinical presentation within the frontotemporal lobar degeneration (FTLD) spectrum disorders and represents a leading cause of early-onset dementia, typically affecting individuals under 65 years of age [1,2]. Clinically, this syndrome is characterized by progressive alterations in cognition, behavior, socioemotional functioning, and personality [3,4], resulting in significant impairments across multiple domains of daily living [5,6]. These changes profoundly disrupt partnerships, parenthood, social interactions, and occupational functioning, severely compromising patients’ quality of life, while placing considerable strain on their families, leading to significant caregiver stress and burden [7].

In comparison to other FTLD clinical subtypes and other types of dementia, such as Alzheimer’s disease (AD), individuals with bvFTD tend to exhibit faster rates of functional decline and more pronounced limitations across various activities of daily living (ADLs) [8,9]. Notably, the majority of these patients develop severe functional impairment within 5 years of symptom onset [10]. However, there is a considerable variability in the rate of disease progression and functional deterioration among individuals with bvFTD [11]. This heterogeneity poses significant challenges for clinical prognostication, particularly in a syndrome that primarily affects the middle-aged population. Therefore, uncovering the determinants of functional impairment in this clinical group is essential for advancing our understanding of the underlying disease mechanisms and improving clinical management and intervention strategies [5,6,12].

### 1.1. Background

Previous studies have identified a broad spectrum of factors associated with functional decline in individuals with bvFTD [5]. These factors include demographic characteristics (such as older age at symptom onset [12] and a positive family history of neurodegeneration [11]), neural parameters (including frontal [12,13] and insular brain atrophy [14], elevated neurofilament light chain levels [15], and dysfunction in inhibitory and facilitatory intracortical circuits as measured by transcranial magnetic stimulation [16]), clinical signs (such as parkinsonism and frontal release signs [11]), genetic mutations (such as, progranulin mutation [12] and C9orf72 expansion [11]), cognitive impairments (including decline in global cognition [11,17,18], executive dysfunction [6,12,19,20], memory impairment [11,19], deficits in language [12], visuospatial abilities [12], and processing speed [19]), difficulties in social cognition [20,21], behavioral symptoms (such as apathy [6,17,20,22,23,24], stereotypic and compulsive behaviors [11,23], disinhibition [20], hallucinations and anxiety [19]), as well as motor symptoms (such as swallowing difficulties [25]).

Specifically, from a cognitive perspective, decline in global cognitive functioning [11,17,18] and deficits in frontal-lobe-related processes [6,12,19,20], such as attentional and executive dysfunction, have been consistently associated with functional impairment and poorer real-world performance in individuals with bvFTD, given their central role in goal-directed behavior, planning, and adaptive problem-solving. Although memory difficulties have also been linked to functional limitations in bvFTD [11,19], evidence suggests that these difficulties are more likely attributable to impairments in executive mechanisms that support the strategic deployment of memory, rather than to a primary degradation of memory storage processes. Specifically, individuals with bvFTD often struggle to sustain attention to episodic details, as well as to initiate memory search and flexibly access, retrieve, and apply episodic information or semantic knowledge in context, according to current goals and situational demands [26,27]. Additionally, although impairments in language and visuospatial abilities have also been identified in some studies as contributing to functional decline in bvFTD [12], such findings are reported less frequently in the literature and are generally considered less robust predictors of functional impairment, compared to deficits in attentional and executive control domains. Finally, social cognition deficits—a hallmark feature of bvFTD—have been associated with poorer performance in ADLs [20,21], as they significantly disrupt individuals’ ability to maintain close interpersonal relationships and navigate complex social interactions.

In addition to cognitive deficits, behavioral disturbances have also been consistently identified as significant drivers of functional impairment in individuals with bvFTD, with greater symptom severity closely associated with more pronounced functional disability [19,20]. Among these disturbances, negative behavioral symptoms, such as apathy, emotional blunting, loss of initiative, and lack of insight, have emerged as particularly strong predictors of functional decline due to their profound impact on motivated goal-directed behavior and everyday autonomy [6,17,20,22,23,24]. These symptoms severely compromise an individual’s capacity to initiate, sustain, and adapt behavior in response to environmental demands, resulting in reduced participation in daily activities, diminished responsiveness to external cues, and accelerated cognitive deterioration due to decreased engagement with cognitively and socially stimulating experiences. Apathy, in particular, has been consistently highlighted as one of the most robust behavioral predictors of functional impairment in bvFTD, often presenting early in the disease course and exerting a detrimental impact on patients’ independence and quality of life [6,17,20,22,23,24]. Positive behavioral symptoms, such as disinhibition, impulsivity, stereotyped behaviors, anxiety, and hallucinations, have also been associated with poorer functional outcomes in bvFTD [11,19,20,23]. These symptoms may significantly impair social judgment and appropriateness, disrupt interpersonal relationships, compromise safety, and interfere with the ability to perform everyday activities. However, their predictive value appears less consistent across studies, and they are generally less strongly linked to functional decline than negative symptoms.

Another domain that is particularly affected and prominently featured in the clinical presentation of bvFTD is personality. Notably, despite personality changes being a core and early hallmark of bvFTD [3], the role of personality traits in predicting functional outcomes has received limited empirical attention in this clinical population. To date, no studies have directly examined whether specific patterns of personality traits are associated with the severity of functional impairment in individuals with bvFTD. This represents a critical gap in the literature, particularly considering the profound impact of personality alterations on emotional processing and interpersonal functioning in this clinical syndrome. Emerging evidence suggests that higher levels of conscientiousness and lower levels of neuroticism are associated with more favorable functional outcomes [28,29,30]. Conscientiousness in particular, defined by characteristics such as competence, orderliness, dutifulness, achievement striving, self-discipline, and deliberation, has been consistently identified across studies as a potential protective factor against cognitive and functional decline in later life [28,30,31,32]. However, whether such associations extend to bvFTD remains unknown and warrants systematic investigation.

In parallel with cognitive and neuropsychiatric factors, neuroimaging research has also provided important insights into the neural underpinnings of functional impairment in bvFTD. Neuroimaging studies investigating the structural correlates of functional disability in bvFTD have consistently demonstrated that the extent and distribution of cerebral atrophy are closely associated with patients’ functional status [12,13,14]. Specifically, an increasing number of studies suggest that individuals exhibiting predominantly frontal or frontotemporal patterns of atrophy tend to experience more severe impairments in both basic and instrumental activities of daily living (BADLs and IADLs, respectively), as well as a more rapid trajectory of functional decline, compared to those with temporally dominant or more posterior atrophy profiles [12,13]. Voxel-based morphometry analyses have further shown that reduced gray matter volume in bilateral, particularly right-sided, frontotemporal regions is significantly associated with greater functional impairment [13]. Notably, despite the growing recognition of these structural markers, complementary neural indicators, such as regional cerebral blood flow (rCBF), have not yet been systematically examined in relation to functional impairment in bvFTD, leaving the brain perfusion correlates of functional decline largely unexplored. However, it is worth mentioning that several recent studies have applied single-photon emission computed tomography (SPECT) imaging at the Brodmann area (BA) level in bvFTD using NeuroGam™ software for the analysis of regional perfusion, providing valuable insights into the neural correlates of the disease. These studies have shown that BA-level SPECT can aid in the differential diagnosis between FTD and AD, distinguish among FTD variants, and identify specific regions involved in particular bvFTD clinical manifestations—including anosognosia and neuropsychiatric symptoms, such as apathy and eating disorders—thereby contributing to a better understanding of their neural underpinnings [33,34,35,36]. Importantly, although these studies investigate perfusion abnormalities at the BA level in bvFTD, to our knowledge, none have systematically investigated the relationship between BA-level perfusion and functional status in this clinical population, highlighting a critical gap that the present study aims to address.

Although the precise mechanisms through which neural, cognitive, socioemotional, and neuropsychiatric deficits collectively contribute to functional impairment in bvFTD remain unclear, existing evidence suggests a directional sequence that parallels those observed in other neurodegenerative syndromes. In particular, progressive cerebral degeneration—especially in the frontal and anterior temporal cortices [37]—appears to initiate the cascade of functional decline commonly observed in individuals with bvFTD. These neuropathological changes are thought to disrupt key cognitive domains such as attention, executive control, and social cognition and contribute to the emergence of behavioral disturbances and personality alterations. However, it is not yet fully elucidated whether cognitive symptoms precede behavioral manifestations or vice versa, as these domains often interact dynamically and may co-emerge. According to cognitive-behavioral models [38], cognitive impairments may contribute to the emergence of behavioral disturbances by undermining regulatory and evaluative processes that guide socially appropriate behavior and emotional functioning [6]. Together, these disruptions compromise an individual’s ability to plan, initiate, regulate, and effectively perform everyday activities, ultimately leading to significant functional impairment. Understanding the directionality of these relationships is essential for constructing targeted models of disease progression and for identifying intervention points to slow or mitigate functional decline.

### 1.2. Goals and Hypotheses of the Study

The present study aims to identify the strongest correlates of functional status in individuals with bvFTD, by investigating the relative contributions of cognitive deficits, behavioral disturbances, personality changes, and brain perfusion abnormalities. Based on existing literature, the following research hypotheses were formulated, in accordance with this first goal:

**Hypothesis** **1.1.** *Lower global cognitive functioning, as well as impairments in frontal-lobe-related processes, such as attentional and executive control abilities, will represent the strongest cognitive correlates of functional impairment in individuals with bvFTD, compared to other measures of cognitive functioning.*

**Hypothesis** **1.2.** *Deficits in theory of mind abilities will be strongly associated with functional impairment in individuals with bvFTD.*

**Hypothesis** **1.3.** *Negative behavioral symptoms, such as apathy and emotional flatness, will be more strongly associated with functional decline than other behavioral manifestations.*

**Hypothesis** **1.4.** *Higher levels of conscientiousness will be associated with more preserved functional abilities in individuals with bvFTD, whereas other personality traits will show no significant association with functional status.*

**Hypothesis** **1.5.** *Hypoperfusion in prefrontal brain regions and in areas functionally connected to the prefrontal cortex will be strongly related to functional impairment in bvFTD.*

Additionally, this study seeks to develop a theoretical framework to elucidate the possible pathways through which these diverse factors interrelate and contribute to functional disability. Specifically, it intends to examine which specific aspects of the disease most significantly impact performance in everyday activities, as well as to explore how these different aspects may interconnect to shape unique functional profiles. The following hypotheses were formulated regarding this second goal:

**Hypothesis** **2.1.** *Hypoperfusion in prefrontal regions will be significantly associated with cognitive deficits in attention and executive control.*

**Hypothesis** **2.2.** *Cognitive deficits will be predictive of more severe behavioral disturbances.*

**Hypothesis** **2.3.** *Behavioral disturbances will be linked to personality changes.*

**Hypothesis** **2.4.** *This sequential pattern, from brain hypoperfusion to cognitive deficits, behavioral symptoms, and personality changes, will collectively predict the degree of functional disability in bvFTD.*

## 2. Materials and Methods

### 2.1. Participants

In this prospective observational study, 26 patients meeting the International Behavioral Variant FTD Criteria Consortium (FTDC) revised guidelines [3] for the diagnosis of at least possible bvFTD, along with their caregivers, were recruited from the 2nd Neurology Clinic of “AHEPA” University General Hospital of Thessaloniki in Greece, between January 2023 and November 2024. Diagnoses were established by a multidisciplinary team of neurologists and neuropsychologists, following comprehensive evaluations. As part of the diagnostic process, all participants underwent thorough clinical and neuropsychological assessments, as well as neuroimaging, genetic testing, and cerebrospinal fluid biomarker analyses to exclude alternative diagnoses. 

To be eligible for inclusion in this study, patients were required to have a close relative or friend who knew them well and could reliably report on their behavior, personality, and functional status. Additionally, to ensure inclusion of individuals in the early stages of bvFTD, only patients with symptom onset within three years prior to enrollment were considered eligible, with symptom duration determined based on informant reports.

Exclusion criteria comprised the presence of major vascular lesions on magnetic resonance imaging (MRI) or computed tomography (CT), which could confound the interpretation of neuroimaging findings, as well as any other neurological conditions (e.g., stroke, Parkinson’s disease, multiple sclerosis, epilepsy, or traumatic brain injury) that could account for functional impairment. Patients with significant psychiatric comorbidities, including schizophrenia, bipolar disorder, or major depression, were also excluded. In addition, individuals with severe sensory or motor impairments, or other physical disabilities that could substantially interfere with the assessment of everyday activities, were considered not eligible.

### 2.2. Procedures

Participants were evaluated during scheduled outpatient visits at the memory clinic, with all assessments conducted in accordance with standardized protocols to ensure consistency and reliability. Each participant was typically assessed across three separate sessions, each lasting approximately 45 min, to minimize fatigue and maintain optimal cognitive performance. During these sessions, patients completed a comprehensive neuropsychological battery to evaluate multiple domains of cognitive functioning. Testing sessions were conducted in quiet, well-lit rooms, free from external distractions to optimize participants’ focus and comfort. In addition, to minimize fatigue, all neuropsychological sessions took place during the morning hours.

Additionally, each patient underwent SPECT imaging to evaluate rCBF across lobes and BAs. The SPECT scans were conducted within a maximum time range of 2 weeks from the neuropsychological assessment to ensure temporal consistency between measures.

Caregivers were also interviewed during scheduled clinic visits, typically across two sessions, each lasting approximately two hours. Semi-structured interviews were conducted to gather detailed information regarding the patients’ behavioral and personality changes, as well as their functional status and level of performance in everyday activities, through the administration of informant-facing questionnaires. In addition, disease staging scales were utilized to assess overall disease severity.

All assessments were conducted by certified clinical neuropsychologists with extensive experience in the dementia field. The full battery of face-to-face neuropsychological tests and informant-based scales will be described below.

#### Protocol Approvals and Patient Consents

The study was reviewed and approved by the Bioethics and Ethics Committee of Aristotle University of Thessaloniki (AUTh), Greece (protocol code: 331191/2022, approval date: 20 December 2022). All patients and their informants were provided with detailed information regarding the nature, objectives and procedures of the research and gave their written informed consent to participate and share data. All data collected were anonymized to protect participant confidentiality, in full compliance with the European Union’s (EU) General Data Protection Regulation (GDPR; Regulation (EU) 2016/679 of 27 April 2016) concerning the processing and protection of personal data [39]. Additionally, all procedures were conducted in accordance with the ethical standards of the Declaration of Helsinki [40].

### 2.3. Neuropsychological Assessment

This section provides an overview of all psychometric tools included in the study.

Global cognitive functioning was assessed using the Montreal Cognitive Assessment (MoCA) [41]. The psychometrically validated Greek version was used [42], with total scores (0–30) analyzed, where higher scores indicate better performance.

The Clock Drawing Test (CDT) [43] was also employed as a measure of global cognitive functioning. The version administered has been psychometrically validated in the Greek population [44,45]. Performance was scored on a 15-point scale, with higher scores reflecting better overall cognitive performance.

The Taylor Complex Figure Test [46] was used as a constructional memory task to assess visuospatial abilities and visual memory. For the purposes of the present study, two variables were included in the analyses: the total score from the copy condition (range: 0–36), reflecting perceptual organization and visuoconstructional skills, and the total score from the delayed recall condition (range: 0–36), reflecting episodic long-term visual memory.

To assess long-term episodic verbal memory, two tests were administered: the Word Learning Test, which evaluates memory for semantically unrelated material, and the Story Memory Test, which assesses memory for semantically related content [45]. For both tests, only the delayed recall scores were used (0–10 and 0–16, respectively). These were summed to create a composite verbal memory index (range: 0–26), with higher scores indicating better performance.

The Confrontation Naming Test [45,47] was used to assess noun retrieval and word-finding abilities. The total number of correctly named items (0–40) was used as an index of naming ability, with higher scores indicating better performance.

The Trail Making Test (TMT)—Part A [48] was employed to assess visual attention and visuo-motor processing speed. The adapted and psychometrically validated Greek version of the test was administered [49,50]. Performance was measured by completion time, with shorter times indicating better performance.

The Forward Digit Span Task [45,51] was used to assess short-term verbal memory and auditory attention. Performance was recorded as the maximum number of digits correctly recalled in order (span score), ranging from 0 to 9, with higher scores indicating better performance.

The selection of executive function measures in the present study was mainly guided by the widely accepted theoretical model proposed by Miyake et al. (2000) [52], which conceptualizes executive functioning as comprising three core components: set-shifting, working memory/updating, and inhibition.

The psychometrically validated Greek version of the TMT—Part B [48,49,50] was used to assess set-shifting and cognitive flexibility. Performance was scored based on completion time, with shorter times indicating a better ability.

The Backward Digit Span Task [45,51] was used to assess auditory working memory. Performance was recorded as a span score ranging from 0 to 8, with higher scores indicating better performance.

The Stroop Test [53] was administered to evaluate inhibitory control, specifically the ability to suppress prepotent (automatic) responses in favor of more appropriate ones. The Greek adaptation of the test, which has demonstrated good psychometric properties, was used in the present study [54].

The Verbal Fluency Test [51] was administered to evaluate an individual’s ability to retrieve specific information under constrained search parameters. In the present study, a psychometrically validated Greek adaptation of the test was utilized [55]. The test comprises semantic and phonemic fluency tasks. In both tasks, higher scores, defined as the total number of valid words generated, indicate better performance.

The FRONTIER Executive Screen (FES) [56], a brief 10–15 min battery originally developed to differentiate FTD from AD, was used to screen for executive impairment. The culturally adapted and psychometrically validated Greek version of the test was administered [57]. The FES evaluates three key executive domains affected in bvFTD through subtests of phonemic verbal fluency, verbal inhibitory control, and verbal working memory. Each subtest is scored 0–5, with a total score ranging from 0 to 15; higher scores indicate better executive function.

In addition to paper-based neuropsychological measures for the assessment of executive functioning, the present study also utilized the online version of the REMEDES for Alzheimer-Revised (R4Alz-R) battery [58,59,60], a computerized tool assessing working memory, attentional control, and executive functioning. Subtest scores were normalized and combined into two composite scores based on R4Alz-R’s validated two-factor structure [59], which were then summed to produce a total executive performance score used in analyses, with higher scores indicating more errors and poorer performance.

As regards social cognition, the Greek adaptation [61] of the Emotion Evaluation Test (EET), which constitutes Part 1 of the Awareness of Social Inference Test—Short (TASIT-S) [62], was employed to assess participants’ ability to accurately recognize basic emotions based on paralinguistic cues. Scores range from 0 to 10, with higher scores indicating better emotion recognition ability.

The Greek adaptation [63] of the Test of Social Inference—Minimal (SI-M), which constitutes Part 2 of the TASIT-S [62], was administered to evaluate participants’ ability to infer others’ mental states in both sincere and sarcastic communicative contexts. The total score, ranging from 0 to 36, reflects the number of correct responses, with higher scores indicating better theory of mind performance.

The Greek adaptation of Goldberg’s International Personality Item Pool (IPIP) Big-Five Questionnaire [64] was utilized to assess patients’ personality traits across five dimensions, in accordance with the Big Five theory [65]: Extraversion-Introversion, Agreeableness, Conscientiousness, Emotional Stability-Neuroticism, and Intellect/Openness. The IPIP has been previously used in dementia research, including studies with FTD populations, and appears to be a suitable instrument for evaluating personality in dementia [66,67,68]. The Greek version of the IPIP has been translated, adapted, and psychometrically validated for use in the Greek population and demonstrates good psychometric properties [69]. Given the limited self-awareness often observed even in the early stages of dementia [70,71], and the particularly pronounced loss of insight characteristic of bvFTD [3,72,73], patients’ self-reports of personality are generally considered unreliable. Although informant-based assessments may be influenced by factors such as the caregiver-patient relationship or subjective perceptions, they are widely regarded as the most valid approach for evaluating personality in individuals with dementia [74]. Therefore, in the present study, the questionnaire was administered to knowledgeable informants. Accordingly, all questionnaire items were rephrased to a third-person format, and informants rated the patients’ current personality traits. Composite scores (10–50) were calculated for each trait, with higher scores indicating greater expression. All five trait scores were used in the analyses.

At the level of behavioral assessment, the Frontal Behavioral Inventory (FBI) [75], a 24-item informant-based questionnaire, was used to quantify the presence and severity of behavioral symptoms typically associated with FTD [76]. The FBI has been extensively used in FTD research and demonstrates very good psychometric properties, including high internal consistency and inter-rater reliability [6,77,78,79,80]. Beyond its research applications, it is also frequently employed in clinical practice to aid in the differential diagnosis between FTD and AD [77,78,79,80]. For this study, the Greek adaptation of the FBI was employed, which has been psychometrically standardized and validated for use with the Greek population [77]. The FBI includes two subscales capturing negative behaviors (e.g., apathy, inflexibility) and positive behaviors (e.g., disinhibition, impulsivity). Each subscale yields a score from 0 to 36, with higher scores indicating more severe symptoms. Both subscale scores were included in the analyses.

The Clinical Dementia Rating (CDR) [81] was used to evaluate disease severity based on cognitive and functional performance across six domains, generating both a Global Score (CDR-GS; 0–3) and a Sum of Boxes score (CDR-SB; 0–18). To enhance sensitivity to the FTLD symptom profile, the FTLD-Modified CDR (FTLD-CDR) [82,83,84] was also employed. This version includes two additional domains—language and behavior—and yields a composite Sum of Boxes score (0–24). For both instruments, higher scores reflect greater overall impairment.

The Greek version of the Frontal Rating Scale (FRS) [83,85], a 30-item informant-based questionnaire, was also employed as an FTD-specific disease staging tool to assess behavioral and functional abilities. The FRS raw score, with a maximum of 30, was converted to a percentage score, with higher values reflecting milder functional and behavioral impairment. This percentage was included in the present study’s analyses.

The Greek version [6,86] of the Disability Assessment for Dementia (DAD) [87] was used as the primary outcome measure to assess functional status based on performance in activities of daily living. This informant-based 40-item scale evaluates both BADLs and IADLs, along with subcomponents of initiation, organization/planning, and task execution. Scores are expressed as percentages, with higher values indicating greater functional independence.

The Greek adaptation of the Functional Activities Questionnaire (FAQ) [88] was utilized as an additional psychometric tool to assess functional status, specifically targeting difficulties in IADLs. Item scores are summed to yield a total ranging from 0 to 30, with higher scores indicating more pronounced functional impairment.

At this point, it is important to note that the DAD was selected as the primary outcome measure in the present study due to its comprehensive assessment of both BADLs and IADLs, thereby enabling a global evaluation of patients’ functional status. The DAD has been extensively validated and widely applied in dementia research, including numerous studies involving bvFTD populations [6,8,17,18,22,23,24]. By contrast, the FRS and FAQ were included as secondary measures in the current study to provide complementary functional insights, as both scales primarily assess IADLs and do not adequately capture BADLs.

### 2.4. Neuroimaging: Brain Perfusion SPECT Scans

Brain perfusion was assessed at the Second Academic Nuclear Medicine Department of “AHEPA” University General Hospital of Thessaloniki, Greece, using SPECT imaging to measure rCBF in lobes and BAs, following EANM guidelines [89]. Participants underwent scanning 30 min after intravenous tracer administration of 740 MBq Hexamethyl Propylene Amine Oxime labeled with Technique-99 m (99mTc-HMPAO SPECT), while at rest. Images were acquired using a Philips gamma camera and reconstructed with filtered back projection. NeuroGam™ scanning data analysis software (Segami-Corporation, Columbia, SC, USA) enabled automated rCBF quantification in lobes and BAs, standardized to Talairach space and compared with an age-matched normative database. Associations between rCBF patterns and functional status were examined to investigate brain perfusion-related contributions to daily functioning in bvFTD. For the analyses of the present study, we used rCBF z-scored patient values against an age-matched control group, thereby accounting for age-related effects on brain perfusion and ensuring comparability across participants. 

More detailed descriptions of the neuropsychological and neuroimaging assessments can be found in the Supplementary Material File S1.

### 2.5. Statistical Analyses

Descriptive statistics were computed using IBM SPSS Statistics V.30 (https://www.ibm.com/us-en, accessed on 7 January 2025) to summarize participants’ baseline demographic and clinical characteristics. Additionally, data were examined for outliers using standardized z-scores and visual inspection of boxplots, with extreme values assessed for potential exclusion. A *p*-value < 0.05 was initially considered the threshold for statistical significance. To account for multiple comparisons, the Bonferroni correction was applied by dividing the significance level (α = 0.05) by the number of variables assessed (25 variables), resulting in an adjusted significance level of 0.002. In all analyses, statistical significance was then determined using this adjusted threshold to control the family-wise error rate (FWER) and reduce the risk of Type I errors.

To identify the strongest correlates of functional impairment in individuals with bvFTD, we first employed a penalized Least Absolute Shrinkage and Selection Operator (LASSO) regression analysis to perform variable selection, allowing us to identify the key correlates of patients’ functional status. Following this, path analysis was conducted to explore the relationships among the most significant variables and their pathways to functional impairment.

In the penalized LASSO regression model, only variables demonstrating a strong correlation with the primary outcome measure—the DAD score—were included as potential predictors. Specifically, only variables exhibiting a statistically significant correlation at *p* < 0.01 (**) were considered for inclusion. This variable reduction strategy aimed at simplifying the subsequent regression model by focusing on those variables most strongly associated with functional status.

LASSO regression analysis was conducted in R programming language and statistical computing environment (version 4.4.3, www.r-project.org, accessed on 20 January 2025) [90] using the “glmnet” package, with the alpha parameter set to 1 for L1 regularization. LASSO, a widely used technique in predictive modeling and machine learning, was chosen for its ability to effectively handle high-dimensional data by shrinking non-informative coefficients to zero, thus enabling variable selection [91,92,93]. The dataset was randomly split into training (70%) and test (30%) subsets to train the model and evaluate its performance. The optimal regularization parameter (λ) was identified through a 10-fold cross-validation process using the cv.glmnet function, minimizing test mean squared error (MSE). The final model, refitted with the optimal λ, was then used to generate predictions, and the resulting regression coefficients were extracted and visualized, allowing for the identification of the most significant predictors of ADL performance.

To further strengthen our findings and ensure the robustness of the final model, we decided to apply the same variable selection procedure to two additional outcome measures commonly used to assess disease severity and functional status in dementia: the FRS and the FAQ scales [11,19,21,94]. This step was undertaken to assess whether the identified predictors of functional status were consistent across different measures of functional abilities, thereby reinforcing the reliability of the final regression model.

Subsequently, path analysis was conducted using EQS version 6.4 [95] to examine the directional relationships among the key predictors identified through the penalized LASSO regression model and the DAD score. Specifically, Structural Equation Modeling (SEM) on covariance matrices was used. Given the extremely small sample size, a series of separate path models was tested to systematically examine and compare the directional relationships among the significant variables [96]. For model confirmation, a non-significant chi-square test (χ^2^, *p* > 0.05) was considered indicative of a good fit, suggesting that the specified model adequately represents the observed data. The Root Mean Square Error of Approximation (RMSEA) was used to assess the model’s approximation error, with a value ≤0.05 suggesting a good fit, while values between 0.06 and 0.08 were considered acceptable and indicated reasonable approximation error. The Comparative Fit Index (CFI) compared the fit of the hypothesized model to the null model, with values ≥0.95 indicating a good fit, and values ≥0.90 considered adequate. Finally, the Standardized Root Mean Square Residual (SRMR) was evaluated, with values <0.05 indicating minimal residual error, and values <0.08 reflecting an acceptable fit.

## 3. Results

### 3.1. Demographic and Clinical Characteristics

The sample consisted of 26 patients diagnosed with bvFTD, with a nearly equal distribution of 12 males and 14 females. The mean age of the participants was 70.19 years (SD = 7.72), with a mean age at disease onset of 68.08 years (SD = 7.85). In terms of educational background, participants had a mean education level of 10.65 years (SD = 3.84). Notably, among the 26 patients included in the study, two carried a genetically confirmed mutation associated with the development of bvFTD, specifically the C9orf72 expansion.

Clinical assessments revealed a mean CDR Global score of 1.17 (SD = 0.68) and a mean CDR Sum of Boxes score of 6.60 (SD = 3.88), indicating mild dementia [97]. As anticipated, the FTLD-CDR Sum of Boxes score was higher, with a mean of 8.60 (SD = 4.41), reflecting greater impairment in areas specifically associated with FTD. As for the type of knowledgeable informants who provided information on the patients, most of them were spouses (65%), followed by adult children (31%), and siblings (4%).

### 3.2. Selection of Variables for Inclusion in the Penalized LASSO Regression Analysis

Out of all variables assessed, the following nine met the inclusion criterion described in the Section 2: performance on the Semantic Verbal Fluency task, the Negative Symptoms subscore from the FBI scale, the Conscientiousness trait score from the IPIP scale, the MoCA total score, the completion time for the TMT—Part A, the copy condition score from the Taylor Complex Figure test, the total performance score from the R4Alz-R battery, the Clock Drawing test score, and the total score on Part 2 of the TASIT-S test. Notably, prior to model fitting, multicollinearity among the selected candidate predictors was evaluated using the Variance Inflation Factor (VIF) values. No evidence of problematic multicollinearity was detected, as all VIFs remained within acceptable limits.

### 3.3. Results of the Penalized LASSO Regression Analysis

**Figure 1:**
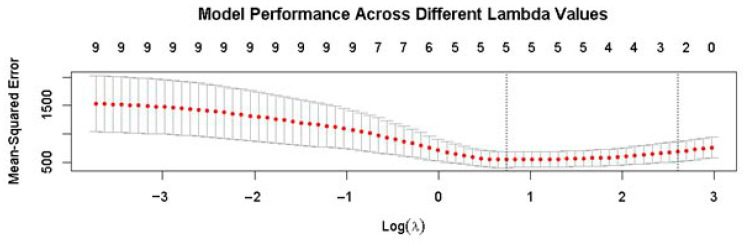
Cross-Validation Plot Showing Model Performance Across Different Lambda Values and the Optimal Lambda Selection. The x-axis represents log(λ), where λ is the LASSO regularization parameter. The y-axis shows the mean-squared error (MSE).

The optimal regularization parameter (λ) identified through the 10-fold cross-validation process was 2.1, which minimized the test MSE. A cross-validation plot (Figure 1) illustrates the model’s performance across various λ values, showcasing the optimal λ that yielded the lowest MSE.

**Figure 2:**
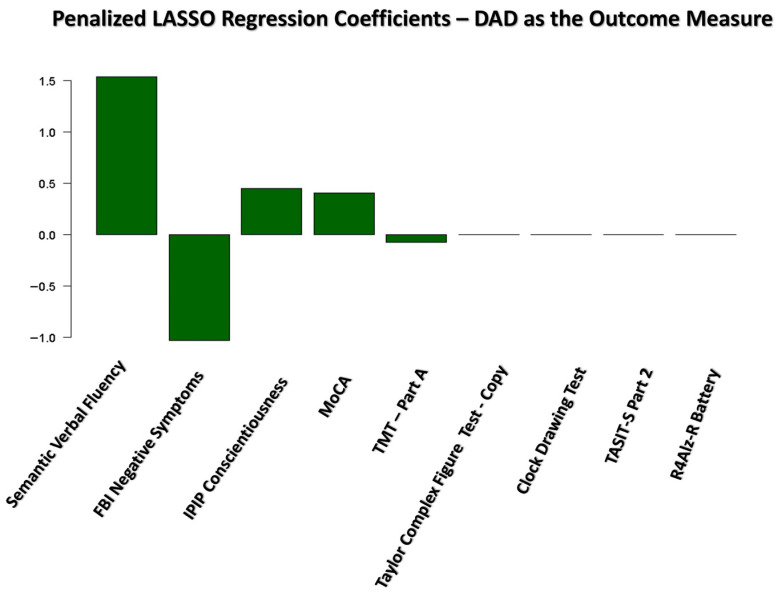
Bar chart illustrating the coefficient estimates of the predictors included in the final penalized LASSO regression model for the DAD total score. Predictors are presented in descending order based on the absolute values of their relative contribution to the model. Abbreviations (in alphabetical order): FBI: Frontal Behavioral Inventory; IPIP: Goldberg’s International Personality Item Pool Big-Five Questionnaire; MoCA: Montreal Cognitive Assessment; R4Alz-R: REMEDES for Alzheimer—Revised battery; TASIT-S: The Awareness of Social Inference Test—Short Form; TMT: Trail Making Test.

The final LASSO regression model identified the following five variables as significant predictors of the DAD score: Semantic Verbal Fluency (β = 1.54), Negative Symptoms, as measured by the FBI scale (β = −1.04), Conscientiousness, as measured by the IPIP scale (β = 0.45), MoCA (β = 0.40), and TMT—Part A (β = −0.08) (see Figure 2). The model’s intercept was estimated at 62.57. The remaining candidate predictors were excluded from the final model, as their coefficients were shrunk to zero, indicating no significant contribution to the prediction.

The final LASSO regression model yielded an MSE of 117.79, indicating the average squared difference between the observed and predicted values. Additionally, the model explained approximately 66.2% of the variance in the outcome variable, with an *R*^2^ value of 0.66.

**Figure 3:**
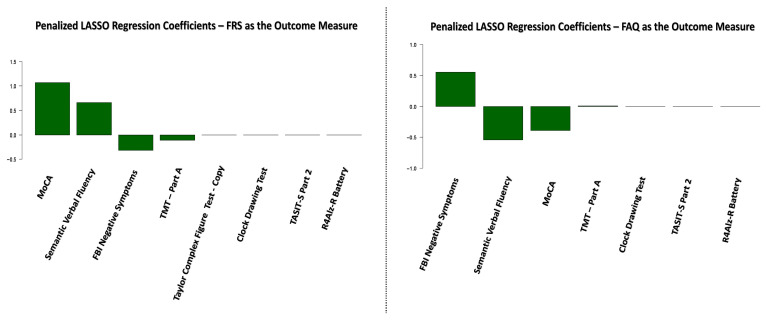
Bar charts depicting the coefficient estimates of the predictors included in the penalized LASSO regression models for the FRS and FAQ scales. Abbreviations (in alphabetical order): FBI: Frontal Behavioral Inventory; MoCA: Montreal Cognitive Assessment; R4Alz-R: REMEDES for Alzheimer—Revised battery; TASIT-S: The Awareness of Social Inference Test—Short Form; TMT: Trail Making Test.

As described in the Section 2, we subsequently applied the same variable selection procedure to two additional outcome measures: the FRS and FAQ scales. The final LASSO regression coefficients for each scale are presented in Figure 3.

As shown in Figure 2 and Figure 3, four predictors—Negative Symptoms, as measured by the FBI scale, Semantic Verbal Fluency, MoCA, and TMT-Part A—were retained as significant predictors across all three LASSO regression models for the DAD, FRS, and FAQ scales. Although the magnitude of the coefficients varied across models, these four predictors consistently contributed to the explained variance in functional abilities. Notably, the Conscientiousness trait, as measured by the IPIP scale, was not included in the FRS and FAQ models, as it did not demonstrate a significant correlation with either scale, and was therefore excluded before entering the LASSO regression analyses.

### 3.4. Brain Perfusion Contributions to Functional Status in bvFTD

Out of the 26 patients included in the study, SPECT data were unavailable only for one participant, resulting in a final sample size of 25 observations. To explore the relationships between rCBF and functional status in early-stage bvFTD, we first generated a correlation matrix in SPSS to examine potential associations between the DAD score and brain lobes, as well as specific BAs in both the left and right hemispheres.

In our sample, the DAD score demonstrated statistically significant correlations with two BAs: BA 8 and 40, both in the right hemisphere. Specifically, moderate positive correlations were observed between the DAD total score and BA 8 (r = 0.41, *p* < 0.05), as well as BA 40 (r = 0.42, *p* < 0.05). In contrast, no significant relationships were observed between functional status and the brain perfusion levels in other BAs or in any lobes.

To confirm the validity of the observed relationships between brain perfusion in BA 8 and 40 of the right hemisphere and functional status, we examined whether these associations also extended to other clinically relevant variables. Beyond the DAD score, we assessed the relationship between brain perfusion in these areas and disease severity measures, including the FTLD-CDR and the FRS. This analysis aimed to establish the external validity of our initial findings, ensuring that the association between rCBF in BA 8 and 40 and functional status was not confined to a single measure but reflected broader disease progression and impairment. Notably, both FTLD-CDR and FRS demonstrated statistically significant relationships with perfusion in right BA 8 and 40, further reinforcing the robustness of this finding. Specifically, perfusion in BA 8 showed a significant correlation with the FRS score (r = 0.51, *p* < 0.01) and the FTLD-CDR Sum of Boxes (r = 0.54, *p* < 0.01). Similarly, BA 40 perfusion was significantly associated with both the FRS percentage (r = 0.42, *p* < 0.05) and the FTLD-CDR Sum of Boxes (r = 0.65, *p* < 0.05). Notably, the strength and significance of the associations observed between BA 8 and disease severity measures were greater than those for BA 40, as reflected in both the correlation coefficients and the lower *p*-values, suggesting that perfusion in BA 8 may be particularly relevant to disease severity and progression in bvFTD.

It is noteworthy that none of the brain perfusion variables met the more stringent, pre-established inclusion criterion of *p* < 0.01 in their correlations with the DAD score and were therefore not included in the previously conducted LASSO regression analysis. Nonetheless, their incorporation into subsequent path analyses was considered scientifically meaningful. Establishing robust associations between brain perfusion and informant-based functional measures is inherently challenging due to methodological complexities. Consequently, even moderate associations at the *p* < 0.05 level merit consideration, particularly when they are theoretically grounded and supported by prior empirical findings. Among the identified regions, BA 8 in the right hemisphere was chosen as the primary brain perfusion variable for the path analyses.

### 3.5. Path Analysis Results

Based on the aforementioned results and the existing literature on the relationships among the variables of interest, we proceeded to run a series of path analyses. Several path combinations failed to meet the required fit indices for validation. The confirmed models are presented below.

**Figure 4:**
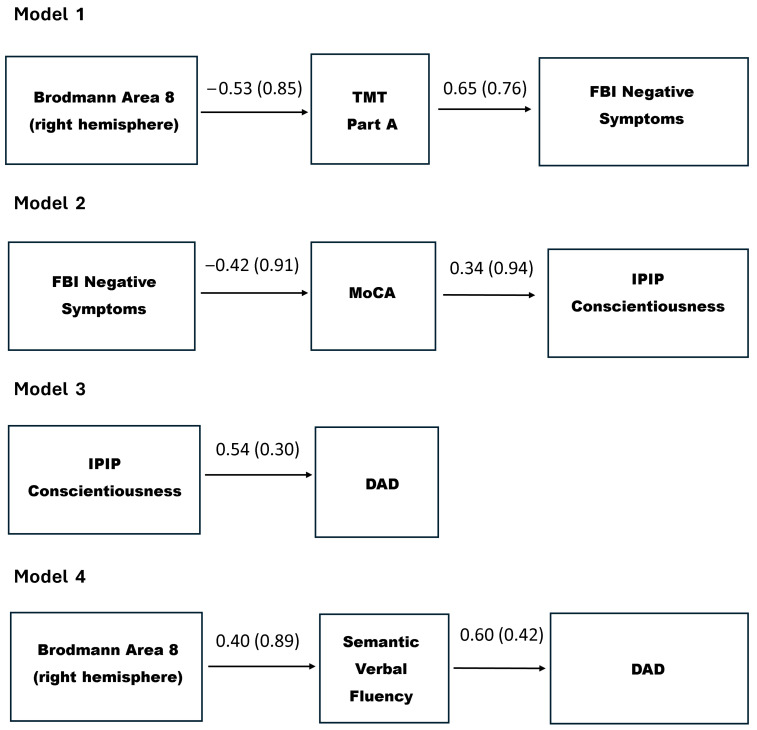
Confirmed path analysis models illustrating the relationships among neural, cognitive, behavioral, and personality-related correlates of functional status in bvFTD. Standardized path coefficients (β) are provided above the arrows, with measurement errors indicated in parentheses. Abbreviations (in alphabetical order): DAD: Disability Assessment for Dementia; FBI: Frontal Behavioral Inventory; IPIP: Goldberg’s International Personality Item Pool Big-Five Questionnaire; MoCA: Montreal Cognitive Assessment; TMT: Trail Making Test.

We first tested a model hypothesizing a pathway from BA 8 in the right hemisphere to TMT-Part A, and subsequently to FBI Negative Symptoms. This model was confirmed with an excellent fit: χ^2^(1, N = 26) = 0.793 (*p* = 0.373), RMSEA = 0.000 (90% CI: 0.00 − 0.51), CFI = 1.000, and SRMR = 0.047 (see Figure 4).

Subsequently, a model hypothesizing a pathway from FBI Negative Symptoms to MoCA and then to IPIP levels of Conscientiousness was confirmed: χ^2^(1, N = 26) = 1.025 (*p* = 0.311), RMSEA = 0.032 (90% CI: 0.00 − 0.52), CFI = 0.996, and SRMR = 0.070 (see Figure 4). Additionally, a direct path linking IPIP Conscientiousness to the DAD score was identified (β = 0.544, *p* < 0.001).

Finally, another model, with a totally different pathway from BA 8 in the right hemisphere to Semantic Verbal Fluency and then to DAD percentage was also confirmed with good fit indices: χ^2^(1, N = 26) = 1.181 (*p* = 0.277), RMSEA = 0.080 (90% CI: 0.00 − 0.55), CFI = 0.987, and SRMR = 0.053 (see Figure 4).

Finally, to evaluate the potential impact of the two participants carrying C9orf72 expansions, given their possible phenotypic differences, we conducted a sensitivity analysis by repeating all of the above statistical analyses after excluding these cases. Importantly, the results remained consistent across both the penalized LASSO regression and the path analyses, indicating that the inclusion of C9orf72 carriers did not substantially influence the study’s findings.

## 4. Discussion

The primary objective of this study was to identify the most significant factors associated with functional impairment in individuals diagnosed with bvFTD, by examining the relative contributions of cognitive deficits, behavioral disturbances, personality changes, and brain perfusion patterns. Beyond identifying key correlates of everyday functioning, this research also sought to construct a theoretical framework to explain how these domains may interrelate and contribute to the observed decline in bvFTD patients’ capacity to perform ADLs.

The findings of this study partially support the proposed hypotheses regarding the most robust contributors to functional impairment in bvFTD. Hypothesis 1.1, which predicted that lower global cognitive functioning and frontal-lobe-related processes, such as attentional and executive control abilities, would be the strongest cognitive correlates of functional decline, was largely confirmed. Specifically, within the cognitive domain, three variables emerged as uniquely predictive of functional status: semantic verbal fluency, MoCA, and TMT-Part A. Among these, semantic verbal fluency stood out as the strongest individual predictor. Semantic cognition refers to the ability to access, apply, and extend knowledge acquired over the lifespan to support a wide variety of both verbal and non-verbal behaviors [98]. A growing body of literature underscores the pivotal role of semantics in supporting real-world functional capacities in dementia, including orientation, communication, financial management, and transportation [21,99,100], suggesting that intact semantic networks are foundational for the effective execution of everyday tasks. In addition, semantic memory has also been found to mediate the relationship between episodic memory and performance on ADLs [99]. Semantic systems provide the conceptual scaffolding necessary for interpreting environmental cues (e.g., understanding task demands) and generating contextually appropriate responses, capacities that are necessary for maintaining autonomy in daily living. The finding that semantic fluency was the most robust predictor of functional status in bvFTD can also be interpreted within the framework of the “Controlled Semantic Cognition” (CSC) model [98,101], which posits that semantic cognition arises from the dynamic interaction of two core components: a “semantic representation” system and a “semantic control” system. The representation system—primarily associated with the anterior temporal lobes—encodes and maintains conceptual knowledge, while the control system—largely supported by prefrontal regions—modulates the retrieval, selection, and manipulation of semantic information in a goal-directed and context-sensitive manner. Within this dual-system architecture, disruptions in either component, or in the interaction between them, can compromise individuals’ ability to maintain, access, and translate semantic knowledge into purposeful behavior in daily life, thereby undermining effective real-world functioning.

The MoCA also demonstrated significant predictive utility. As a global cognitive screening tool, its inclusion in the final model is in line with prior research supporting its broad clinical applicability in bvFTD [102] and highlighting the relevance of sensitive broad cognitive screening tools for capturing functional impairment in bvFTD [11,17,18].

Similarly, TMT-Part A, a task assessing visual attention and psychomotor processing speed, was also retained in the final model. Despite its procedural simplicity, this task proved to be a sensitive marker of functional variation, suggesting that even basic attentional and processing speed capacities may serve as foundational capacities for everyday functioning. This finding also highlights the potential value of using simple, brief, and accessible neuropsychological tools in clinical settings to monitor functional decline.

In contrast, several initially correlated cognitive measures did not demonstrate independent predictive utility. For instance, theory of mind measures did not emerge as significant predictors in the final model. Therefore, Hypothesis 1.2, which proposed that deficits in theory of mind abilities would be strongly associated with functional impairment in individuals with bvFTD, was not supported by the present findings. One possible explanation is that, while theory of mind is an important aspect of social cognition, other unmeasured constructs, such as socioemotional sensitivity or real-world empathy, may be more closely tied to functional outcomes, and these domains were not directly assessed in the current study.

As expected, traditional memory and language assessments—domains often emphasized in the context of other neurodegenerative conditions, such as AD—did not show significant associations with functional status in this cohort. This finding is not unexpected and is consistent with the typical neuropsychological profile of bvFTD, in which memory and core language functions are relatively preserved in the early disease stages.

Beyond cognitive predictors, both conscientiousness and negative behavioral symptoms were retained in the final model as significant contributors, confirming Hypotheses 1.3 and 1.4, and reaffirming the central role of behavioral and personality changes in shaping everyday functioning in bvFTD. Conscientiousness refers to a personality dimension reflecting organization, discipline, impulse control, deliberation, consideration of consequences, achievement striving, and goal-oriented behavior [64,65,103,104]. In our sample, bvFTD individuals with higher levels of conscientiousness exhibited better functional abilities, suggesting that the preservation of this trait may serve as a protective buffer against functional decline. This finding aligns with broader gerontological literature linking conscientiousness to healthier neural, cognitive, and functional aging trajectories [28,30,31,32]. Notably, the other Big Five personality dimensions did not show significant predictive value in this sample, underscoring the unique relevance of conscientiousness in the bvFTD functional phenotype.

The severity of negative behavioral symptoms, such as apathy, emotional flatness, and loss of insight, also emerged as a robust predictor of functional status in bvFTD. This finding is consistent with existing literature, which highlights the malignant impact of negative behavioral symptoms, such as apathy, on functional outcomes in bvFTD [5,6,17,20,22,23,24]. These behavioral disturbances have been consistently linked to diminished initiation and behavioral motivation, all of which are critical for maintaining independence in daily life and engagement in daily activities. In contrast, positive behavioral symptoms, such as disinhibition, did not show strong relationships with functional status in this sample, suggesting that motivational impairments may have a more direct and disabling impact on daily life than impulsive behaviors.

Importantly, this study also investigated the neuroimaging correlates of functional impairment in bvFTD, by examining rCBF using brain SPECT imaging. In line with hypothesis 1.5, the results revealed that hypoperfusion in BAs 8 and 40, both located in the right hemisphere, was significantly associated with greater functional disability. Notably, reduced perfusion in these brain regions was also strongly correlated with indicators of clinical disease severity, including the FRS and the FTLD-CDR scales, highlighting these two regions as key neural correlates of disease progression.

Right BA 8, located in the superior frontal gyrus of the prefrontal cortex, is involved in key higher-order cognitive processes, including attention regulation, uncertainty-driven decision making, motor planning, and behavioral regulation [105,106,107,108,109,110]. These functions are critical for initiating and sustaining goal-directed behavior, which is essential for daily functioning and autonomy. In addition, BA 8′s caudal portion (area 8A) includes the frontal eye fields, which play a central role in oculomotor control and visuospatial processing, particularly in initiating large-amplitude saccades and selecting between competing environmental visual stimuli. Its involvement in both the default mode and central executive networks positions it as a key integrative hub supporting flexible cognitive and behavioral control [105,106].

Right BA 40, located in the supramarginal gyrus of the inferior parietal lobe, plays a crucial role in integrating multimodal somatosensory input with higher-order cognitive and socioemotional functions, including language, semantic processing, self-awareness, self-reflection, and the regulation of attention to social and emotional cues [111,112,113]. Highly interconnected with prefrontal regions and embedded within broader cortical networks, BA 40 supports dynamic interactions between cognitive control and emotional regulation, processes essential for functional autonomy. Its involvement in the integration of multimodal somatosensory information also suggests that its dysfunction could significantly impair an individual’s ability to carry out routine tasks, due to somatoperceptual deficits, impairments in spatial processing, and disrupted sensorimotor coordination, ultimately contributing to a remarkable decline in functional independence.

Taken together, these rCBF findings suggest that right BA 8 and 40 may represent critical neural targets for future research in bvFTD, potentially providing valuable insights that could further unravel the complex neurobiological mechanisms underlying the disease.

**Figure 5:**
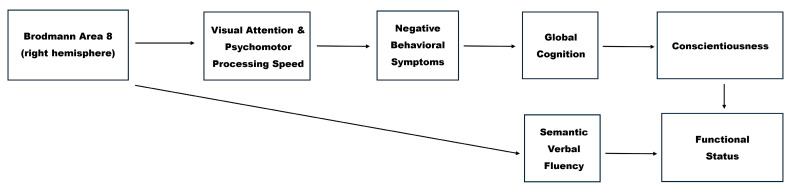
Proposed theoretical model illustrating the interrelationships among cognitive, behavioral, personality, and brain perfusion correlates of functional status in individuals with bvFTD.

Building on the outcomes of the confirmed path models, we advanced to develop a theoretically grounded and statistically informed explanatory model, offering a preliminary framework for a deeper understanding of the underpinnings of functional impairment in bvFTD (see Figure 5). This model delineates the interrelationships among brain perfusion abnormalities, cognitive deficits, behavioral disturbances, and personality changes, highlighting their converging contributions to real-world functional decline. According to the proposed model, and as illustrated in Figure 5, the observed pattern of directional relationships provides preliminary and partial support for Hypotheses 2.1 through 2.4. Specifically, hypoperfusion in right BA 8 appears to be associated with attentional and processing speed deficits, which in turn are linked to more pronounced negative behavioral symptoms. These behavioral disturbances are further related to declines in global cognition and conscientiousness, ultimately correlating with poorer daily functioning.

Therefore, while the overall sequence outlined in the hypotheses was supported, the findings revealed a more nuanced trajectory than initially anticipated. Rather than a broad pattern in which cognitive deficits uniformly precede behavioral disturbances, the results suggest that specific cognitive processes, particularly attention and speed, are disrupted early and may serve as catalysts for the emergence of negative behavioral symptoms, which, in turn, appear to contribute to a secondary decline in global cognitive functioning. This refined understanding highlights the dynamic interplay between different domains in shaping the functional phenotype of bvFTD.

At the foundation of the proposed model lies the well-established principle that the clinical manifestations of bvFTD, like those in other dementing syndromes, are driven by progressive cerebral degeneration [37,114,115,116,117,118]. Accordingly, the cascade of disruptions identified in our model begins with neural alterations—specifically, hypoperfusion in right BA 8. Our data confirmed strong and widespread associations between this brain region and disease severity measures, rendering it a compelling entry point for modeling the downstream effects on everyday functioning. Its functional relevance in the current model is supported initially by its significant association with performance on the TMT-Part A, a task that involves visual scanning, attentional shifting, motor planning, and psychomotor speed [119]. BA 8 is critically involved in guiding the allocation of spatial attention and selecting between competing visual stimuli based on conditional rules [105,106,119]. The link between hypoperfusion in right BA 8 and impaired TMT-Part A performance provides a neuroimaging basis for the attentional dysfunction often observed in the early stages of FTD [119,120]. This finding also aligns with neurocognitive models emphasizing the particular vulnerability of attention to neural disruptions, like hypoperfusion, due to its dependence on widely distributed brain networks [121]. Because attention relies on such extensive and interconnected neural systems, it is especially susceptible to early dysfunction across various neurological disorders.

The proposed model advances to demonstrate that impairments in visual attention, along with processing speed deficits, significantly contribute to the severity of negative behavioral symptoms. These symptoms, characterized by diminished emotional expressiveness and responsiveness, reduced initiation of behavior, and a general flattening of affect and volition, are clinically recognized as core features of the apathetic presentation in bvFTD [3]. Attentional impairment and psychomotor slowing are likely to impair patients’ ability to flexibly allocate attentional resources, formulate internal goals, and initiate actions, resulting in a behavioral profile marked by indecisiveness, emotional flatness, and reduced spontaneity.

Importantly, these negative behavioral disturbances appear to contribute to subsequent declines in global cognitive functioning. Our findings align with previous research indicating that negative behavioral symptoms are associated with lower performance in cognitive tasks [122,123]. This pattern also supports the hypothesis of a feedback loop in which diminished behavioral activation and social withdrawal limit cognitive engagement, thereby accelerating the neurodegenerative process and functional deterioration. Prior studies have identified apathy—a prominent negative symptom affecting over 90% of people with bvFTD [124]—as a potent predictor of overall cognitive decline in bvFTD, potentially due to reduced stimulation and decreased activation of the underlying brain networks.

In the proposed model, the decline in global cognition is, in turn, associated with reductions in the levels of conscientiousness, a personality dimension encompassing traits such as organization, diligence, self-discipline, and persistence [64,65,103,104]. Our findings suggest that this trait degradation is not a primary personality change, but rather a secondary consequence of the accumulating neural, cognitive, and behavioral impairments. As cognitive dysfunction and negative symptoms worsen, patients gradually lose the capacity to structure their daily lives, regulate behavior according to internal goals, and maintain consistency in task execution. This decline may also reflect a compromised ability to simulate future outcomes and diminished insight into the importance of goal-oriented behaviors, undermining conscientious functioning and, ultimately, impinging on ADL performance.

Beyond the sequence described above, verbal fluency emerged as another critical cognitive domain linked to hypoperfusion in right BA 8, representing an additional pathway associated with functional decline in bvFTD. The timed nature of the task accentuates its executive demands, particularly in relation to initiation and sustained mental effort. Interestingly, previous research suggests that semantic fluency deficits in bvFTD are primarily driven by executive control dysfunction, rather than by degradation of semantic knowledge per se, as typically observed, for example, in semantic dementia [27]. Supporting this view, neuroimaging studies using FDG-PET have shown that semantic verbal fluency performance in bvFTD correlates with metabolism in frontal regions, rather than in temporal areas [27]. Consistent with these findings, the present study identified a significant association between semantic verbal fluency and hypoperfusion in BA 8, a prefrontal region critically involved in executive control [106]. Prefrontal cortex disruptions may compromise individuals’ capacity to strategically deploy semantic content, even when underlying conceptual knowledge remains relatively intact, particularly in the early stages of the disease. Thus, from the perspective of the CSC framework outlined earlier [98,101], semantic verbal fluency deficits in bvFTD may primarily reflect a breakdown in the top-down “semantic control” system, disrupting patients’ ability to effectively initiate retrieval and flexibly access, organize, and navigate semantic networks and internal representations, ultimately contributing to remarkable difficulties in everyday functioning. For instance, a patient with bvFTD may know the concept of “fruits” and recognize individual items like “apple” or “banana”, but when asked to rapidly generate a list of fruits, they might struggle to initiate search and sustain retrieval, producing only a few examples or mentioning unrelated words. This difficulty potentially does not stem from loss of semantic knowledge, but rather from impaired executive control that hinders the strategic search and organization of relevant information, making everyday tasks—such as planning a grocery list, accessing and applying social norms in interpersonal contexts, or engaging in meaningful conversations—particularly challenging.

The cumulative impact of the above-mentioned interrelated disruptions culminates in substantial functional disability in bvFTD. Importantly, the conceptual model proposes that these deficits result from a cascading sequence of dysfunctions, rather than isolated impairments. This underscores the need for clinical assessments and interventions that consider the dynamic interdependence between brain function, cognitive control, behavioral motivation, and personality in shaping functional trajectories in bvFTD.

### 4.1. Clinical Implications

The findings of the present study carry important implications for both the clinical assessment and management of individuals with bvFTD. First, by identifying which standardized measures best correlate with real-world functional abilities, this research offers valuable evidence for the ecological validity of various neuropsychological tools in this clinical population. The comprehensive neuropsychological battery employed in this study allowed for an exploration of the relationship between cognitive performance and functional status, yielding useful information on which measures most accurately predict everyday functioning [125,126,127]. This understanding is crucial for refining clinical assessments and ensuring they are not only theoretically sound but also practically relevant and meaningful in the context of real-world outcomes for individuals with bvFTD.

Additionally, the proposed combination of predictive measures—including negative behavioral symptoms on the FBI, the conscientiousness trait on the IPIP, semantic verbal fluency, MoCA, and TMT-Part A—constitutes a clinically useful and time-efficient neuropsychological toolkit for assessing individuals with bvFTD. The estimated administration time of this battery ranges from 30 to 45 min, with 45 min representing the upper limit required for completion. Importantly, only three of the tasks (MoCA, TMT-Part A, and the semantic verbal fluency task) are administered directly to the patient and take about 15 min in total, while the remaining measures are addressed to the caregiver and are completed within a similarly brief timeframe. Although administering the full battery may not always be feasible in routine clinical practice, its use, when possible, can provide valuable insights into the severity of symptoms and the degree of functional impairment, thereby informing individualized care.

Moreover, the variables identified in this study hold significant promise as valuable tools for tracking disease progression over time. Within this framework, these measures can also be utilized as potential endpoints in longitudinal studies or clinical trials [128,129,130], helping to monitor disease trajectory and evaluate the effectiveness of therapeutic interventions. However, it is essential that these variables be validated longitudinally in larger and more diverse cohorts to confirm their long-term clinical utility.

Finally, by identifying the key neural, cognitive, behavioral, and personality factors underlying functional impairment in bvFTD, this study highlights areas where tailored interventions can be implemented and lays the groundwork for the development of more targeted, refined, and personalized intervention strategies for the clinical management of affected individuals. These insights can guide therapeutic efforts, inform individualized care planning, and support targeted rehabilitation approaches aimed at slowing functional deterioration and enhancing patients’ quality of life [131,132,133,134,135,136,137,138]. By integrating this knowledge into routine clinical practice with bvFTD, our findings provide a foundation for more effective, patient-centered care, with the potential to translate into meaningful improvements in daily functioning and overall well-being.

### 4.2. Limitations

A key limitation of the present study is the relatively small sample size (n = 26), which may affect both the reliability of the statistical models and the generalizability of the findings. This constraint partly reflects the low prevalence of bvFTD, which inherently restricts the available pool of participants. Furthermore, our focus on a clinically homogeneous group of individuals with early-stage disease, intended to minimize confounding factors, further narrowed the sample. Additionally, the stringent inclusion criteria, which excluded participants with comorbid medical conditions, also contributed to the limited sample. The small sample size may have reduced the statistical power of our analyses, hindering the detection of subtler associations and increasing the uncertainty in parameter estimates, potentially affecting the stability and generalizability of the predictive models. Consequently, these findings should be considered preliminary and interpreted with caution. Future research with larger and more diverse samples is needed to validate and extend these observations and to provide more robust and reliable estimates.

Additionally, the cross-sectional design of this study precludes any conclusions about causality or the temporal evolution of the observed associations. Relationships among brain perfusion, personality, cognitive and behavioral factors, as well as functional status were assessed at a single time point, without capturing the dynamic, progressive nature of bvFTD. Future research should therefore prioritize longitudinal designs [139,140,141,142,143,144] to investigate how the identified factors dynamically interact over the course of the disease. Such studies could elucidate how individual functional trajectories are shaped and inform the development of prognostic models, thereby helping to identify individuals at higher risk of faster disease progression who may require enhanced clinical support [145,146,147,148,149,150].

Furthermore, while this study employed a comprehensive set of neuropsychological tools, it did not assess several key dimensions of social cognition, such as empathy and socioemotional semantic knowledge, which are highly relevant in bvFTD [21]. These aspects are likely to play a significant role in functional impairment, particularly in bvFTD, where social and interpersonal deficits are prominent [3]. Future research should aim to incorporate these socioemotional variables to provide a more complete understanding of the role of social cognition in functional decline [151].

Finally, this research did not account for the potential impact of genetic factors, comorbidities, or treatment effects on functional status. Including these additional variables could offer a more comprehensive view of the observed relationships and further enrich our understanding of the disease [152,153,154].

### 4.3. Strengths and Unique Contribution

This study’s main strength lies in its multifactorial and integrative design, which enabled a comprehensive assessment of various factors associated with functional impairment in early-stage bvFTD. By incorporating these factors within a unified, theoretically grounded explanatory framework—a multidimensional perspective rarely employed in previous research on this population—this study provided valuable insights into how distinct disease features affect real-world functioning. The extensive neuropsychological assessment systematically covered a wide range of domains, while functional abilities were evaluated using multiple standardized tools, enabling validation of predictive models across different measures of everyday functioning. Moreover, a further strength lies in the study’s direct investigation of personality traits, which, despite being a core and early hallmark of bvFTD, have received limited empirical attention in relation to functional outcomes in this clinical population. Finally, the integration of neuropsychological findings with rCBF neuroimaging data allowed for a more nuanced exploration of brain-behavior relationships. While structural imaging has been widely used in prior research, no studies to date have employed rCBF measures to investigate neuroimaging correlates of functional impairment in bvFTD.

## 5. Conclusions

Hypoperfusion in key prefrontal and parietal regions, along with the subsequent cognitive and neuropsychiatric manifestations, appears to be associated with the pronounced functional limitations observed in individuals with bvFTD, even in early stages. The findings of this study not only validate the intricate and multifaceted nature of functional impairment in bvFTD, but also underscore the importance of adopting a multidimensional, integrative approach to elucidate the complex mechanisms underlying functional decline in this clinical population. Rather than operating independently and at a general level, specific neural, cognitive, behavioral, and personality-related factors appear to interact dynamically, collectively shaping the severity of real-life functional disability. Specifically, the severity of negative behavioral symptoms, levels of conscientiousness, and performance on neuropsychological measures of semantic verbal fluency, visual attention, visuomotor speed, and global cognition were identified as the strongest correlates of performance in ADLs. Understanding the key determinants of the disease can inform the development of targeted, personalized treatment strategies and care approaches aimed at mitigating functional deterioration and improving patient outcomes.

In conclusion, the findings of this study not only advance our theoretical understanding of bvFTD but also offer tangible, clinically relevant tools and strategies. Incorporating these findings into clinical practice could inform patient assessment and management, support clinical decision making, and guide the development of refined, person-centered therapeutic interventions. Future research should build on this work by replicating findings in larger and more diverse cohorts, employing longitudinal designs to track neural, cognitive, behavioral, and personality trajectories over time, and integrating neuroimaging and biomarker assessments to better understand the mechanisms underlying functional decline in bvFTD [155,156,157,158,159,160,161,162,163,164,165,166]. These efforts hold great promise for informing prognostic models, optimizing patient management, and enhancing personalized interventions and treatment strategies, ultimately translating into improved care and quality of life for affected individuals [167,168,169,170].

## Data Availability

Anonymized data may be shared upon request to the corresponding author from any qualified investigator for the purpose of replicating procedures and results.

## Supplementary Materials

The following supporting information can be downloaded at: https://www.mdpi.com/article/10.3390/jpm15100466/s1. Supplementary Material File S1. Detailed Descriptions of the Neuropsychological and Neuroimaging Assessments.

## Abbreviations

The following abbreviations, listed in alphabetical order, are used in this manuscript:

AD	Alzheimer’s disease
ADLs	Activities of daily living
AUTh	Aristotle University of Thessaloniki
BA	Brodmann area
BADLs	Basic activities of daily living
bvFTD	Behavioral variant frontotemporal dementia
CDR	Clinical Dementia Rating Scale
CDT	Clock Drawing Test
CFI	Comparative Fit Index
CSC	Controlled Semantic Cognition model
CT	Computed tomography
DAD	Disability Assessment for Dementia
EANM	European Association of Nuclear Medicine
EET	Emotion Evaluation Test
EU	European Union
FAQ	Functional Activities Questionnaire
FBI	Frontal Behavioral Inventory
FES	FRONTIER Executive Screen battery
FRS	Frontotemporal Dementia Rating Scale
FTDC	Frontotemporal Dementia Criteria Consortium
FTLD	Frontotemporal Lobar Degeneration
FTLD-CDR	Frontotemporal Lobar Degeneration-Modified Clinical Dementia Rating Scale
FWER	Family-wise error rate
GDPR	General Data Protection Regulation
IADLs	Instrumental activities of daily living
IPIP	Goldberg’s International Personality Item Pool Big-Five Questionnaire
LASSO	Penalized Least Absolute Shrinkage and Selection Operator regression analysis
MoCA	Montreal Cognitive Assessment
MRI	Magnetic Resonance Imaging
MSE	Mean Squared Error
rCBF	Regional cerebral blood flow
RMSEA	Root Mean Square Error of Approximation
R4Alz-R	REMEDES for Alzheimer-Revised battery
SD	Standard deviation
SEM	Structural equation modeling
SI-M	Social Inference-Minimal Subtest
SPECT	Single Photon Emission Computed Tomography
SRMR	Standardized Root Mean Square Residual
TASIT-S	The Awareness of Social Inference Test—Short Form
TMT	Trail Making Test
χ^2^	Chi-square test
99mTc-HMPAO	Hexamethyl Propylene Amine Oxime labeled with Technique-99 m
