# Supplementary material for: Functional Impairment in Behavioral Variant Frontotemporal Dementia: Cognitive, Behavioral, Personality, and Brain Perfusion Contributions
Source: J Pers Med. 2025 Oct 1;15(10):466. doi: 10.3390/jpm15100466 (PMC12565498; doi:10.3390/jpm15100466)
Supplement: Supplementary file 1 [file jpm-15-00466-s001.zip › jpm-3814690-supplementary.pdf]

Supplementary Material

Neuropsychological Assessment

Cognitive Assessment

Global Cognitive Functioning

Global cognitive functioning was assessed using the **Montreal Cognitive Assessment (MoCA)** [41], a widely adopted brief screening tool designed to detect mild cognitive impairment and early signs of neurocognitive disorders. The MoCA was selected over other screening tools, such as the Mini-Mental State Examination (MMSE), due to its greater sensitivity to early cognitive changes and its inclusion of tasks that assess executive functioning, a domain commonly affected in the early stages of bvFTD and not adequately captured by the MMSE. The MoCA is a 30-point instrument, typically administered in approximately 10-15 minutes, that evaluates a broad range of cognitive domains, including visuospatial abilities, executive functioning, short-term memory, attention and working memory, language, abstract reasoning, and orientation. For the purposes of the present study, the Greek adaptation of the MoCA was used [42]. The total score, ranging from 0 to 30, was included in the analyses as an index of global cognitive functioning, with higher scores reflecting better overall cognitive performance.

The **Clock Drawing Test (CDT)** [43] is a widely used neuropsychological screening tool for the detection of cognitive impairment, particularly in older adults. In the present study, the version included in the "Neuropsychological Battery" developed by the Laboratory of Cognitive Neuroscience at the School of Psychology, AUTh, was administered to participants [44, 45]. The CDT was employed as a measure of global cognitive functioning, as successful performance on the task involves the integration of multiple cognitive domains. Specifically, performance relies on semantic memory (e.g., knowledge of how a clock looks, what its components are, and how time is represented on it), sustained attention, receptive language, visuo-spatial abilities (e.g., perception of size, distance, and spatial relationships), visuo-constructional skills (e.g., motor programming and visuo-motor coordination), as well as executive functions, (e.g., initiation, planning/organization, and self-monitoring) [44].During the

task, participants are instructed to draw a clock and place the hands to indicate the time “quarter to seven” (6:45). Scoring is based on a 15-point scale, with higher scores indicating better overall cognitive performance.

Visuospatial Abilities and Visual Memory

The **Taylor Complex Figure Test** [46] is a constructional memory task designed to assess visuospatial abilities, perceptual organization, visual episodic short-term and long-term memory, as well as visual recognition. In the present study, three conditions of the test were administered: copy, immediate recall, and delayed recall. In the copy condition, participants were asked to reproduce a complex two-dimensional figure as accurately as possible on a blank sheet of paper. Without prior warning, they were subsequently instructed to draw the same figure from memory, first immediately (immediate recall) and then again after a 20-minute delay (delayed recall). Each of these conditions was timed, with a maximum duration of 5 minutes. Performance was scored based on the accurate reproduction and spatial placement of 18 specific design elements. In all three conditions, higher scores reflect better performance. For the purposes of the present study, only two variables were included in the analyses: the total score for the copy condition (range: 0-36), reflecting visuospatial and visuoconstructional abilities, and the total score for the delayed recall condition (range: 0-36), reflecting long-term visuospatial memory performance.

Verbal Memory

To assess long-term episodic verbal memory, two tests from the “Neuropsychological Battery” [45], developed by the Laboratory of Cognitive Neuroscience at the School of Psychology, AUTh, were administered: the Word Learning Test and the Story Memory Test. The **Word Learning Test** [45] was administered to evaluate memory for semantically unrelated verbal material. This test assesses episodic short- and long-term verbal memory, as well as recognition. It consists of four consecutive trials during which participants are asked to immediately recall a list of 10 semantically unrelated words. Following a delay of approximately 30 minutes, and without prior warning, participants are instructed to recall the list again (delayed recall), followed by a recognition task in which they are asked to identify the target words among distractors.

For the analyses of the present study, only the delayed recall score, ranging from 0 to 10, was considered. The **Story Memory Test** [45] was employed to assess memory for semantically related verbal information. It comprises two trials of immediate recall of a brief story consisting of 16 meaningful items. Following a delay of approximately 30 minutes - during which other non-verbal tasks were administered - participants are asked to recall the story again (delayed recall). For this study, only the delayed recall score, ranging from 0 to 16, was considered. To derive a comprehensive index of long-term episodic verbal memory performance, delayed recall scores from both the Word Learning and Story Memory tests were summed, resulting in a composite score ranging from 0 to 26. Higher scores reflect better performance. This composite score was used in subsequent analyses.

Language

The **Confrontation Naming Test** [47], included in the "Neuropsychological Battery" [45] developed by the Laboratory of Cognitive Neuroscience at the School of Psychology, AUTh, was employed to assess noun retrieval abilities and identify word-finding difficulties (anomia). The version used in the present study consists of 40 black-and-white line drawings depicting objects from a range of semantic categories, including animals, fruits, vegetables, furniture, clothing, tools, and means of transportation. Participants were asked to name each item as accurately as possible. The total score, calculated as the number of correctly named items (range: 0-40), was included in the analyses as an index of confrontation naming ability, with higher scores indicating better performance.

Visual Attention and Processing Speed

The **Trail Making Test (TMT) – Part A** [48] was employed to assess visual attention and visuo-motor processing speed. In the present study, the culturally adapted and psychometrically validated Greek version was administered [49, 50]. TMT-A consists of 25 circles randomly distributed across a sheet of paper, each numbered from 1 to 25. Participants are instructed to draw lines connecting the numbers in ascending order (i.e., 1–2–3…25) as quickly and accurately as possible, without lifting the pencil from

the paper. Scoring is based on the completion time of the task (in seconds), with shorter times indicating better performance.

Auditory Attention and Short-Term Memory

The **Forward Digit Span Task** [51] was used to assess verbal short-term memory and auditory attentional capacity. The version of the task used in the present study is included in the "Neuropsychological Battery" developed by the Laboratory of Cognitive Neuroscience at the School of Psychology, AUTh [45]. During the task, participants listen to sequences of digits and are instructed to repeat them in the exact order presented. In this study, performance was recorded as a span score, defined as the maximum number of digits correctly recalled in sequence. Scores ranged from 0 to 9, with higher scores indicating better performance.

Executive Functions

The selection of executive function measures in the present study was guided by the widely accepted theoretical model proposed by Miyake et al. (2000) [52], which conceptualizes executive functioning as comprising three core components: set-shifting (the ability to flexibly shift between tasks or mental sets), working memory/updating (the capacity to update and monitor working memory representations), and inhibition (the ability to suppress automatic or prepotent responses in favor of more contextually appropriate ones). In addition to these three core components of executive control, the present study also included verbal fluency as an important executive domain, given its reliance on multiple executive processes, including initiation, organization of lexical information, cognitive flexibility, strategic retrieval, and self-monitoring.

To comprehensively evaluate this broad spectrum of executive skills, we employed a multimodal approach that integrated both traditional paper-based neuropsychological tests and a computerized neuropsychological battery, thus combining well-established assessment tools with innovative digital technology.

Traditional (Paper-Based) Executive Function Tests

Set-Shifting

The **TMT – Part B** [48, 49, 50] is a widely used neuropsychological measure for assessing set-shifting and cognitive flexibility. This part of the test consists of 25 circles distributed across a sheet of paper, each containing either a number or a letter. Participants are instructed to connect the circles in an ascending, alternating sequence of numbers and letters (i.e., 1–A–2–B–3–C, etc.), as quickly as possible, without lifting the pencil from the paper. Scoring is based on the total time (in seconds) required to complete the task, with shorter completion times reflecting better performance.

Working Memory – Updating

The **Backward Digit Span Task** [45, 51] was used to assess working memory. During the task, participants are required to repeat a sequence of digits in reverse order, engaging attentional processes, short-term memory, and the mental manipulation of verbal information. In this study, performance was recorded as a span score, defined as the maximum number of digits correctly recalled in reverse sequence. Scores ranged from 0 to 8, with higher scores indicating better performance.

Inhibition

The **Stroop Test** [53] was administered to evaluate inhibitory control, specifically the ability to suppress prepotent (automatic) responses in favor of more appropriate ones. The Greek adaptation of the test, which has demonstrated good psychometric properties, was utilized in the present study [54]. The administered version includes three conditions: the word task, the color task, and the incongruent (color-word) task. For the purposes of this study, emphasis was placed on the incongruent condition, in which participants were presented with a page containing five columns of the color words “red,” “blue,” and “green” printed in mismatched ink colors (e.g., the word “red” printed in blue ink) on a white background. In this condition, participants are instructed to ignore the written words and name aloud the ink color as quickly and accurately as possible, within a 45-second period. This task specifically assesses the ability to suppress automatic word reading in favor of color naming. The total number of correctly named ink colors was used for the present study, with higher scores indicating better inhibitory control.

Verbal Fluency

The **Verbal Fluency Test** [51] is designed to evaluate an individual's ability to retrieve specific information under constrained search parameters. In the present study, the Greek adaptation of the test was administered [55], specifically the shortened version included in the "Neuropsychological Battery" [45] developed by the Laboratory of Cognitive Neuroscience at the School of Psychology, AUTh. The test consists of two parts: the semantic (category) fluency task and the phonemic fluency task. In the semantic fluency task, participants are instructed to generate as many words as possible belonging to a specific semantic category (e.g., animals), as quickly as possible, within a 60-second timeframe. In the phonemic fluency task, they are asked to produce as many words as possible beginning with a designated letter of the Greek alphabet (e.g., X-Chi) within the same time limit. Proper nouns (e.g., names of people or places), lexical derivatives, as well as grammatical variants of previously produced responses (e.g., plurals or verb conjugations) are not accepted as correct answers. These two tasks assess distinct cognitive processes. Semantic fluency primarily relies on intact semantic memory, organization and efficient search processes, whereas phonemic fluency is more strongly associated with cognitive flexibility and shifting abilities [55]. In both tasks, higher scores, defined as the total number of valid words generated, indicate better performance. Sub-scores for each of the two tasks were included in the analyses of the present study.

Executive Screening

The **FRONTIER Executive Screen (FES)** was used as a screening tool for executive impairment [56]. It is a brief executive function battery, typically administered in approximately 10-15 minutes, and was originally developed to distinguish FTD from AD. In the present study, the culturally adapted and psychometrically validated Greek version of the test was administered [57]. The FES assesses three key executive domains that are commonly affected in bvFTD, with three subtests designed to evaluate phonemic verbal fluency, verbal inhibitory control, and verbal working memory. In the verbal fluency subtest, participants are asked to generate as many words as possible within one minute that begin with a given letter; this is performed with two different letters. The verbal inhibitory control subtest uses a sentence completion task that requires participants to inhibit a dominant, automatic verbal

response and instead produce a less expected but appropriate ending (e.g., "The hungry bear found some stale…"). The working memory subtest involves repeating strings of letters in reverse order (e.g., T-K-R must be repeated as R-K-T). Each subtest is scored on a scale from 0 to 5, and the total FES score ranges from 0 to 15, with higher scores indicating better executive functioning.

Computerized Executive Function Assessment

In addition to paper-based neuropsychological measures for the assessment of executive functioning, the present study also utilized the online version of the **REMEDES for Alzheimer–Revised (R4Alz-R) battery** [58-60], an innovative, computerized cognitive assessment tool specifically designed to evaluate three major dimensions of cognitive control abilities: (a) working-memory capacity, (b) higher-order attentional control, and (c) executive functioning, including inhibitory control, task-switching, and cognitive flexibility. The R4Alz-R battery is based on well-established paradigms from cognitive and neuropsychological research and is designed to minimize the influence of educational and cultural biases. For the purposes of the current study, a subset of the R4Alz-R battery was administered to participants. The first component was the Working Memory Capacity Test, which consists of three subtests: (i) the *Short-Term Storage* subtest which assesses the storage component of working memory, with scores ranging from 2, indicating the poorest performance, to 7, representing the best performance, (ii) The *Processing* subtest which evaluates the manipulation of information under working memory demands, using the same scoring range (2–7), and (iii) The *Working Memory Updating* subtest which measures the ability to revise and update stored information, with scores ranging from −58 (reflecting maximum errors) to 14 (indicating optimal performance). The second module administered was the Attentional Control Test, which evaluates various levels of attention, ranging from basic alertness to more complex attentional abilities. This test assesses multiple aspects of attention, including sustained, selective and divided attention. It includes three subtasks with seven levels of increasing difficulty, and performance scores range from 0, indicating the best performance with no errors, to 202, indicating the worst performance with maximum errors. The third component administered was the Executive Functioning Test, composed of two subtests: (i) the

Inhibition subtest which assesses the ability to suppress prepotent responses, with scores ranging from 0, indicating no errors, to 60, indicating the maximum number of errors, and (ii) the *Task/Rule Switching* subtest which evaluates cognitive flexibility and the ability to shift between different task sets or rules, with scores ranging from 0 to 63. The combined score for these two subtests yields a total ranging from 0 (best performance) to 123 (worst performance), reflecting overall performance in both inhibition and switching abilities. Although the full R4Alz-R battery includes additional tasks, such as the Episodic Memory Test, only the subtests described above were administered in the present study to maintain a specific focus on core executive processes. To allow comparability across subtests, normalization of the subtest scores was conducted using z-scores. Subsequently, in accordance with the two-factorial structure identified in previous validation studies of the R4Alz-R battery [59], two composite scores were derived by summing the normalized scores of the subtests associated with each factor. Finally, these two composite scores were aggregated to generate a single total performance score, which was used in the analyses of the present study.

Social Cognition

Emotion Recognition

The Greek adaptation [61] of the **Emotion Evaluation Test (EET)**, which constitutes Part 1 of **the Awareness of Social Inference Test - Short (TASIT-S)** [62], was employed to assess participants' ability to accurately recognize basic emotions based on paralinguistic cues. The test consists of 10 brief video vignettes (approximately 20 seconds each), featuring one or two actors engaged in everyday monologues or dialogues. The verbal content of these scenes is emotionally neutral and contextually ambiguous, with emotional meaning conveyed solely through nonverbal modalities—such as facial expressions, vocal prosody, body posture, gestures, and, in the case of two-person scenes, the interactive responses of the dialogue partner. After viewing each video, participants are asked to identify the expressed emotion by selecting one of seven options: happy, sad, angry, surprised, disgusted (revolted), fearful (anxious), or neutral. The total score, ranging from 0 to 10, reflects the number of correctly identified emotions, with higher scores indicating better emotion recognition ability.

Theory of Mind

The Greek adaptation [63] of the **Test of Social Inference - Minimal (SI-M)**, which constitutes Part 2 of the **TASIT-S** [62] was administered to evaluate participants' ability to infer others' mental states. This subtest assesses individuals' understanding of sincere and sarcastic communicative exchanges, focusing on their capacity to discern a speaker's intended meaning based on visual and vocal paralinguistic cues, including facial expressions, body posture, head and hand gestures, as well as voice prosody. During the task, participants view nine brief video vignettes (each lasting less than one minute) portraying naturalistic social interactions. These vignettes include four sincere and five sarcastic scenarios, with the latter further subdivided into simple and paradoxical sarcastic exchanges. Following each video, participants are asked to answer four yes/no questions targeting their understanding of the characters' actions, thoughts, words, and emotions. The total score, ranging from 0 to 36, reflects the number of correct responses, with higher scores indicating better performance in theory of mind abilities.

Personality Assessment

The Greek adaptation of **Goldberg's International Personality Item Pool (IPIP) Big-Five Questionnaire** [64] was utilized to assess patients' current levels of personality traits. The version employed in the present study consists of 50 items and evaluates the five major personality dimensions in accordance with the Big Five theory [65]: (i) Extraversion-Introversion, (ii) Agreeableness, (iii) Conscientiousness, (iv) Emotional Stability-Neuroticism, and (v) Intellect/Openness. Each trait is represented by 10 items, rated on a 5-point Likert-type scale, ranging from 1 (very inaccurate) to 5 (very accurate). The IPIP has been previously used in dementia research, including studies with FTD populations, and appears to be a suitable instrument for evaluating personality in dementia [66–68]. The Greek version of the IPIP has been translated, culturally adapted, and psychometrically validated for use in the Greek population [69]. The IPIP was selected as the personality assessment tool for this study due to its open-access availability, ease of administration, and the existence of validated scoring keys accessible via the official IPIP website (https://ipip.ori.org/). Given the limited self-awareness often observed even in the early stages of dementia [70,71], and the

particularly pronounced loss of insight characteristic of bvFTD [3,72,73], patients’ self-reports of personality are generally considered unreliable. Although informant-based assessments may be influenced by factors such as the caregiver-patient relationship or subjective perceptions, they are widely regarded as the most valid approach for evaluating personality in individuals with dementia [74]. Therefore, in the present study, the questionnaire was administered to knowledgeable informants. Accordingly, all questionnaire items were rephrased from a first-person format (“I”) to a third-person phrasing (“he/she”). Informants were instructed to evaluate the patients' current personality traits as observed in the present stage of the disease, thereby accounting for any personality changes that have occurred as part of the disease progression. For each personality trait, a composite score ranging from 10 to 50 was calculated in accordance with established scoring keys, with higher scores indicating greater trait expression. All five trait scores were used in the analyses.

Behavioral Assessment

The **Frontal Behavioral Inventory (FBI)** [75] is a 24-item informant-based questionnaire designed to assess and quantify the presence and severity of behavioral symptoms typically associated with FTD [76]. The FBI has been extensively used in FTD research and demonstrates very good psychometric properties, including high internal consistency and inter-rater reliability [6,77–80]. Beyond its research applications, it is also frequently employed in clinical practice to aid in the differential diagnosis between FTD and AD [77–80]. For this study, the Greek adaptation of the FBI was employed, which has been psychometrically standardized and validated for use with the Greek population [77]. The scale is administered through a face-to-face interview with a knowledgeable informant familiar with the patient’s daily behavior, and typically requires 20-30 minutes to complete, depending on the range and severity of the symptoms. The questionnaire comprises two subscales: 12 items assessing negative behaviors associated with deficits (e.g., apathy, aspontaneity, indifference, inflexibility, personal neglect, disorganization, inattention, loss of insight, logopenia, verbal apraxia, loss of comprehension, and alien hand), and 12 items evaluating positive behaviors associated with disinhibition (e.g., perseverations/obsessions, hoarding, excessive jocularity, social inappropriateness, poor judgment and

impulsivity, restlessness/roaming, irritability, aggression, hyperorality/food fads, hypersexuality, utilization behavior, and incontinence). Each item is rated on a 4-point scale: 0 = none, 1 = mild/occasional, 2 = moderate, and 3 = severe/most of the time. For the purposes of the analyses, the two subscale scores for negative and positive symptoms were included, each ranging from 0 to 36, with higher scores reflecting more severe behavioral disturbances.

Disease Severity Measures

The **Clinical Dementia Rating (CDR)** scale [81] was employed to assess the severity of cognitive and functional impairment through a semi-structured interview conducted with both the participant and the reliable informant. The scale evaluates performance across six domains: memory, orientation, judgment and problem solving, community affairs, home and hobbies, and personal care. Each domain is rated on a 5-point scale: 0 = none, 0.5 = questionable (not applicable to the personal care domain), 1 = mild, 2 = moderate, and 3 = severe impairment. Two primary scores are derived from the CDR: the Global Score (CDR-GS) and the Sum of Boxes (CDR-SB). The CDR-GS is calculated using a standardized algorithm and provides an overall staging of dementia severity, ranging from 0 (no dementia) to 3 (severe dementia). The CDR-SB is computed by summing the individual domain ratings, yielding a total score between 0 and 18, with higher scores indicating greater cognitive and functional decline. In addition, the Greek version of the **FTLD-Modified CDR (FTLD-CDR)** scale [82-84] was administered. The FTLD-CDR has been shown to be more sensitive to the FTLD symptomatology than the standard CDR scale [84]. It is an expanded version of the original CDR, assessing two additional domains - language and behavior - resulting in a total of eight domains evaluated. The FTLD-CDR Sum of Boxes (FTLD-CDR-SB) is calculated by summing the ratings across these eight domains, yielding a total score ranging from 0 to 24, where higher scores reflect greater overall impairment.

The Greek version of the **Frontal Rating Scale (FRS)** [83, 85] was also used to assess disease severity in participants, serving as a staging tool specifically designed for FTD. The FRS is effective in characterizing the progression of disease severity and identifying changes in both behavioral and functional abilities over time. The scale consists of 30 items, with a score of 30 representing full functional ability and the absence of

behavioral symptoms, while lower scores indicate a decline in daily functioning and noticeable behavioral changes. After administration, the obtained FRS raw score is divided by the number of applicable items and multiplied by 100 to yield a percentage score. This percentage was included in the analyses, with higher values reflecting milder levels of functional and behavioral impairment.

Functional Measures

The **Disability Assessment for Dementia (DAD)** [87] was used as the primary outcome measure to assess functional status and evaluate patients' performance in activities of daily living (ADLs). For the present study, the Greek adaptation of the DAD scale was utilized [6, 86]. Although originally developed as a functional measure for individuals with Alzheimer's disease, the DAD scale is now widely applied in FTD populations, as well [6,8,17,18,22–24]. It is an informant-based scale consisting of 40 items that evaluate both BADLs and IADLs. Of these 40 items, 17 relate to BADLs and assess fundamental self-care tasks such as hygiene, dressing, continence, and eating. The remaining 23 items pertain to IADLs and evaluate more complex activities such as meal preparation, telephone use, community outings, management of finances and correspondence, medication adherence, and engagement in leisure activities and housework. The DAD also evaluates three key subcomponents of ADL performance: initiation, which refers to the ability to decide on and begin an action; organization/planning, which involves the ability to identify the different components of a task, sequence them appropriately, and prepare the necessary materials; and effective performance/execution, which measures the ability to complete a task in a safe and appropriate manner. To prevent potential gender or cultural bias, the DAD omits questions that are not applicable to the patient's lifestyle or routine. For instance, no points are deducted if cooking was not a part of a patient's daily routine before the onset of their disease. The final score is expressed as a percentage, with scores ranging from 0 to 100, where higher scores indicate better ADL performance and greater functional independence. In the present study, in addition to the total DAD score, sub-scores for BADLs and IADLs were calculated, as well as scores for each of the three ADL performance subcomponents.

The Greek version of the **Functional Activities Questionnaire (FAQ)** [88] was utilized as an additional psychometric tool to evaluate functional status and to quantify difficulties specifically in IADLs. The FAQ is a collateral-report measure comprising 10 items, each rated on a 4-point Likert scale that reflects varying levels of functional independence: 0 = normal, 1 = has difficulty but manages independently, 2 = needs assistance, and 3 = fully dependent. Informants were asked to assess participants' performance over the past 4 weeks in 10 distinct areas of IADLs: (1) writing checks, paying bills, or balancing a checkbook; (2) organizing tax or business records and affairs; (3) shopping independently for clothing, household items, or groceries; (4) engaging in a game of skill or hobby; (5) heating water, making coffee, or turning off the stove after use; (6) preparing a balanced meal; (7) staying informed on current events; (8) paying attention to, understanding, and discussing a TV program, book, or magazine; (9) remembering appointments, family events, holidays, or medications; and (10) traveling beyond the neighborhood, driving, or arranging public transportation. The individual item scores were summed up to produce a total FAQ score, ranging from 0 to 30, with higher scores indicating more severe functional impairment and a greater loss of independence in performing daily activities.

Neuroimaging Assessment: Brain Perfusion SPECT scans

Imaging data acquisition was conducted at the Second Academic Nuclear Medicine Department of the "AHEPA" University General Hospital of Thessaloniki, Greece. Brain perfusion was assessed using SPECT imaging to measure rCBF in lobes and BAs. Imaging procedures, including preparation, patient assessment, and data acquisition were conducted in accordance with the EANM guidelines for brain perfusion SPECT studies [89], ensuring standardized protocols for optimal data quality and consistency. Prior to the assessment, participants were instructed to refrain from alcohol, stimulants (e.g., caffeine), and other substances that could influence rCBF. SPECT scans were performed 30 minutes after the intravenous administration of 740 MBq Hexamethyl Propylene Amine Oxime labeled with Technique-99 m (^{99m}Tc-HMPAO SPECT), while patients were at rest, with their eyes open and ears unplugged. The examination was conducted in a quiet room, minimizing external stimuli or interactions. A single-headed gamma camera (Philips with Pegasys processor) was

used, acquiring 120 projections per participant, with a total scan duration of 40 minutes (20 seconds per projection). Image processing was carried out using the XELERIS Workstation (Version 3.1), employing filtered back projection (FBP) reconstruction with a Butterworth filter (order 10, cutoff 0.5), and reoriented in coronal, sagittal, and transverse planes. NeuroGam™ scanning data analysis software (Segami-Corporation, Columbia, South Carolina, USA) was applied for automated scanning data analysis, enabling rCBF quantification in lobes and BAs for both hemispheres. This software aligns the images within a standardized reference space (Talairach's atlas), enabling comparisons of rCBF SPECT studies across individuals, independent of variations in brain shape, size, or positioning during imaging. Additionally, NeuroGam™ allows comparisons with an age-adjusted normative database of healthy individuals, facilitating the identification of brain perfusion abnormalities. Associations between rCBF patterns and functional status were examined to investigate brain perfusion-related contributions to daily functioning in bvFTD. For the analyses of the present study, we used rCBF z-scored patient values against an age-matched control group, thereby accounting for age-related effects on brain perfusion and ensuring comparability across participants.
